# A Network Theory Approach to Curriculum Design

**DOI:** 10.3390/e23101346

**Published:** 2021-10-15

**Authors:** John O’Meara, Ashwin Vaidya

**Affiliations:** Department of Mathematics, Montclair State University, Montclair, NJ 07043, USA; omearaj1@montclair.edu

**Keywords:** complexity, curriculum, network, education theory, creativity

## Abstract

In this paper we hypothesize that education, especially at the scale of curriculum, should be treated as a complex system composed of different ideas and concepts which are inherently connected. Therefore, the task of a good teacher lies in elucidating these connections and helping students make their own connections. Such a pedagogy allows students to personalize learning and strive to be ‘creative’ and make meaning out of old ideas. The novel contribution of this work lies in the mathematical approach we undertake to verify our hypothesis. We take the example of a precalculus course curriculum to make our case. We treat textbooks as exemplars of a specific pedagogy and map several texts into networks of isolated (nodes) and interconnected concepts (edges) thereby permitting computations of metrics which have much relevance to the education theorists, teachers and all others involved in the field of education. We contend that network metrics such as average path length, clustering coefficient and degree distribution provide valuable insights to teachers and students about the kind of pedagogy which encourages good teaching and learning.

## 1. Introduction

This project aims to utilize the mathematical theory of networks to understand the significance of connectivity in mathematics education. We hypothesize that education is a deeply connected complex system of processes and ideas. Therefore, the role of an effective educator lies in effectively highlighting these features and helping students make their own connections [1]. Such a pedagogy, based on a constructivist philosophy of learning, allows students to personalize learning and strive to be ‘creative’ which essentially amounts to ‘meaning making’ or generating new meaning out of old ideas [2,3,4].

We test our hypothesis by examining the network structure of a precalculus course. Precalculus is an entry-level (and occasionally remedial) course taught in various United States institutions, and populated each semester by a large number of students. The skills taught in this course are essential to most STEM (Science, Technology, Engineering, and Mathematics) disciplines, and therefore a firm grounding in the concepts of precalculus is needed for students to succeed in the sequence of courses that follow. The vast significance and demand for this course has resulted in a flood of textbooks on this subject, with each author and publisher promoting their own philosophy, pedagogy and content. While the content remains similar, different books highlight different topics and come with different expectations and assumptions. While the ensuing discussion will surround the effectiveness of precalculus pedagogy, this article is primarily meant to be an exposition on the importance of a complexity theory-based approach towards curricular design. Education has previously been recognized to be a complex system [5,6,7,8] and it is well articulated in the literature that the tools of complexity theory are very apt to understand critical dynamics and properties of educational systems, be it at any scale. The current work is one ‘micro-scale’ such attempt in the endeavor to understand the complex structure of a curriculum and its implications throughout the entirety of education.

A complexity-based approach to education was perhaps best articulated by William Doll in his numerous works on the subject (see, for instance, [9]). In his article [10] states: “Order emerges from interactions having just the ‘right amount’ of tension or difference or imbalance among the elements interacting”. In the context of education, one could argue that this emergence of order is nothing but creativity. Hiebert and Carpenter [11] ([2006, p. 69]) associate the notion of ‘understanding’ to that of a complex network. They state: “Understanding increases as networks grow and as relationships become strengthened with reinforcing experiences and tighter network structuring”. It has also been argued that learning through identification of similarities and differences between alternate representations of the same information can stimulate the construction of useful connections [2]. Therefore it is quite reasonable to think of education at all levels as an “adaptive and self-organizing complex system”.

This paper is based on the premise that a textbook, which represents a particular pedagogy of the subject being treated, is a collection of multiple interacting parts. As students progress in a course, they navigate from one topic to another, united in the endeavor of comprehending, connecting, and unifying concepts. It is the potential to form their own bridge across different topics that is of significance here, since it relates to creativity:an oft spoken about but understudied phenomenon in higher education. Previous studies on creativity, in mathematics education in particular, have pointed to the importance of making connections as a necessary condition for creativity in the classroom [2,12,13]. The ‘lego model’ of creativity discussed in [2] argues for personalization of knowledge and connection between different ideas which can result in stability and longevity of understanding. The ‘connected curriculum’ program based in UCL [14] espouses the importance of connections in higher education, highlighting the ‘real value’ of education, which lies in preparing students for the ‘real world’ where students must solve complex problems which do not appear under a disciplinary guise. The rationale for such a curriculum is well articulated in [15,16] and other related works, pointing to the fact that “social problems and issues transcend disciplinary boundaries” [16,17]. Our approach in this paper is based on a similar philosophy of learning and clarified using a simpler example, which lends itself to very novel, rigorous and interesting mathematical analysis. We content that such a hybrid modeling approach which combines qualitative and quantitative aspects of mathematics is extremely appropriate in the context of education, especially mathematics education.

Our approach to introducing and evaluating this particular philosophy is as follows: First, we discuss in detail the entire mapping process of mathematizing the curriculum in Section 2. This is followed in Section 3 by the results of our computations. The Section 4 discusses the considerations and results of considering the fitting of each sampled text’s degree distributions, while Section 5 considers how the results from Section 3 might be interpreted with respect to an optimally enriched textbook in which all observed connections are made. In the final section of our paper, we discuss the interpretations of the network-based analysis in the context of curricular design and education, and reflect on the prospects and importance of such an analysis in other problems related to education.

## 2. Complexity and Curriculum

While there is a compelling case for curriculum as a complex system in the literature [9,10,18,19,20,21,22], we feel the need to restate some of these ideas. The term ‘complexity’ itself is vaguely defined and depends on the field to which it is being applied, the central feature of any complex system being that it contains multiple nonlinearly interacting parts. Any new foray into the area of complexity theory therefore requires clear articulation of its structure and evidence that this fundamental feature is met. Davis describes three areas of education where complexity theory has made its mark thus far [19]: (a) “…contents of curriculum, complexity as a disciplinary discourse…”, (b) “…beliefs on learning, complexity as a theoretical discourse…” and (c) “…pedagogical strategies, complexity as a pragmatic discourse…”. The subject matter of this paper falls in the first of these categories.

**Figure 1:**
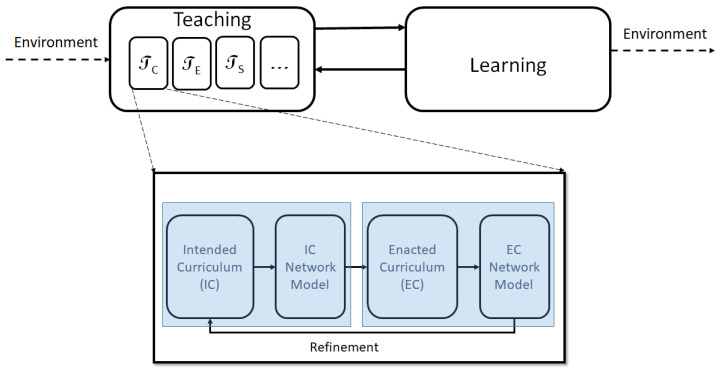
Schematic showing the complex nature of education and curricular design. While teaching and learning are recognized to proceed along an a-causal feedback loop, teaching at its intrinsic scale is feedback between the intended and enacted curriculum, each of which gets refined over time.

Educators recognize the fact that teaching and learning cannot be spoken of independently; they proceed through feedback between each other (see Figure 1). We can think of the curricular aspect of teaching as an interaction between an intended curriculum (IC) and enacted curriculum (EC) (In the Figure 1, IC and EC compartments are mediated by their corresponding network models.). The intended curriculum can be reflected through a textbook which an instructor typically would utilize to design her own lesson plans which is enacted in the classroom, and referred to as the enacted curriculum. The differences between the IC and EC can vary drastically with instructor, students, topic, level of course etc. In this paper, each text chosen is mapped to a unique graph (described in detail below). In mathematical terms, teaching as defined in our study, is the mapping

$$T=T_{C}\cup T_{E}\cup T_{S}\ldots$$

where $T_{C}$ represents the curricular or content aspect of teaching, $T_{E}$ refers to the teaching environment and $T_{S}$ to the skill level of the teacher, among others. We specifically focus on $T_{C}$ which can be represented as the transformation:

$$T_{C}:IC-Model\to EC-Model$$

Based on Figure 1, the map $T_{C}$ can itself be decomposed into several individual components that outline the feedback model generated between IC and EC, and how these steps in the development of curricular content might influence one another. While T is represented as a one-way mapping, in the hands of an experienced instructor, the EC can result in a transformation of the IC as well. It is also not unreasonable to assume, especially in the case of inexperienced instructors, that $T_{C}=I$ (identity map), i.e., the IC is the same as the EC.

It is important to recognize that teaching and learning are embedded within a specific environment, which they are shaped by and influence. Environmental factors directly impact IC (and directly or indirectly, EC), which takes the form of various editions/updates of a book, new books and revisions to course content etc. This interaction between education and it’s various components with the environment maintains education in an *out-of-equilibrium state*, much like a dissipative system [9,23]. If we persist with the thermodynamic language, we can expect this mutual interaction to result in emergent self-organized states, under fixed conditions, which translates to an optimal (meta)stable curriculum (or network pattern) which would change with time and environmental factors. While this argument relies primarily on analogies, it is evident that the educational system is a complex combination of interacting self-similar components, each of which represents a complex system at its own level [20,21,22].

## 3. Mathematical Model

### 3.1. Assumptions

We undertake a network-based analysis of precalculus courses as a means to understand curricular design. Mapping the problem of curricular design to a mathematical framework lends itself to particular forms of analysis which might otherwise not be possible, although the maxim “The Map Is Not the Territory” [24] is relevant and guards against overstating the significance of such a framework. This particular course was chosen since it is a preliminary mathematics course that a large number of students at any institution take and also among the more fundamental courses in mathematics which is required for any further quantitative study, no matter what the discipline. Therefore, inferences drawn about such a course would be of relevance to a large number of students. A fundamental assumption in this study is that the choice of the textbook utilized in the course is a reflection of the teaching philosophy of the instructor, and lays the groundwork for how the course will be conducted, although we do realize that a seasoned instructor can provide meaningful supplements to the course through the introduction of ideas and content not necessarily discussed in the textbook. However, we will assume that the vast majority of precalculus instructors rely quite heavily on the adopted textbook, and so do students in their pursuit to learn the material.

### 3.2. Network Construction

For purposes of this study, we identified several different precalculus textbooks based on their recent popularity as determined by recent adoption of the text in United States universities, and its availability as seen from in-print status on platforms like amazon.com. Older editions of books were still utilized if these were the only ones available to us; we assume that the overall teaching philosophy does not change very much over a few editions of the book, especially if the primary authors of the books remain the same. A list of books adopted for this study is provided in the Table 1.

We examine the underlying network structure of these books (lEach text referenced in Table 1 is encoded carefully by hand to construct its corresponding network using a process defined below.) by identifying connections of the various topics covered in the books. Any two topics are said to be ‘connected’, or ‘linked’, provided there is a sufficiently strong and intentional relationship between them in the text, i.e., the text explicitly refers to one when introducing and/or discussing the other. For example, consider the following line that may appear in a traditional precalculus textbook:

“*One clear distinction that arises between the functions we have studied thus far is that while a linear function exhibits a constant rate of change, an exponential function exhibits a constant percent change*”.

The connections that can be observed in this line are: (a) Linear functions and rate of change, (b) linear functions and exponential functions, (c) exponential functions and percent change and (d) rate of change and percent change. These connections could be meaningfully displayed in tabular format to highlight these connections, as provided below:
**Introduced Topic****Directly Connected Topic**Linear FunctionsRate of ChangeLinear FunctionsExponential FunctionsExponential FunctionsPercent ChangeRate of ChangePercent Change

When encoding lines directly from a text in the above fashion, one might consider whether or not there are inherently connections that exist between every topic introduced when adjoined in the same sentence; in essence, is the set of all elements presented naturally inclined to consist of connections that act as a power set of all elements? This naturally lends itself to the possible construction of a union graph in which each element in a cluster is locally connected to all other elements of that set. While this would certainly yield a higher clustering coefficient, the paths between topics become elongated, less efficient, and far less meaningful-in particular, because edges are being generated based on pure proximity rather than connotation and linguistic intention. In the aforementioned example, it would not be appropriate to connect ‘Linear Functions’ and ‘Percent Change’ because the divisions that are enumerated are between the categories themselves, and all respective highlighted properties. Specifically, we obtain the connection between ‘Linear Functions’ and ‘Exponential Functions’ from the phrase, “between the functions we have studied”, as well as extracting the connection between ‘Rate of Change’ and ‘Percent Change’ from the first few words of the sentence, “One clear distinction”; the former communicates a link between these two particular subtypes of relations that are united by belonging to the class of functions, while the latter describes a contrasting element that inherently differentiates the two. While one is comparative and the other contrastive, describing any given interaction between properties or definitions within the scope of a given subject warrants a meaningful relationship between the two that is intended to bring the topics together in an intentional way. While we are able to find clear links between the remaining two connections by proximity itself, it has been demonstrated that pure proximity itself, and direct mention of relevant topics, is not sufficient. One must also exercise caution and resist adding connections when encoding, as we seek metrics according to the print itself, and not what a teacher of any caliber may supplement. It may seem relevant to ask if such a task is best accomplished by the index of a book. However, it needs to be pointed out that (a) not all books (including the ones we used in this study) had complete indices and (b) indices for books are often done through computer and human effort, with the latter contributing a significant part of such a project [35]. In fact, it is easily noted that not all terms in a book end up in the index [36], and in fact the construction of indices is often left to the ingenuity of the indexer and the purpose/audience of the book.

Naturally, elements of this procedure are somewhat subjective; some instructors may highlight connections others might find unimportant or overlook. However, in the pursuit of developing a more significant understanding of the properties that make a text suitable for a given course, instructor, or student body, it is imperative that causal connections be strictly observed, both in proximity and conceptual basis, revealing an inherently complex nature amongst the association between any given curricular themes.

In our efforts to generate uniform procedure, we conducted an initial trial in which the same text was encoded by two separate researchers, and then results were compared in order to verify that results were not only as identical to one another as possible, but also to ensure that all included nodes and edges were strictly obtained from the textbook at hand, and were not the result of any personal bias or opinion as to which topics might warrant connectivity from the perspective of the researcher; the goal of this encoding process was to establish routine procedure and strict adherence to intentional language dictated by the text, while also taking advantage of the ability for human-based encoding to pick up on nuanced connections similar to the examples outlined in the sample text above.

By regarding each topic as a node and each link as an edge, we are able to meaningfully translate each text into a graph or network of ideas and their corresponding connections. A uniform set of principles were developed to help maintain consistency in mapping the various books. These include:

1. To start with, we make a list of all the topics in the chosen text under each chapter, section, and subsection. These topics are listed in a Table A1 in Appendix A. Each topic is given a numeric code. In the language of sets, $X=\{k_{i}:1<i<N\}$ pertains to the set of N∈N topics covered in the textbook, where $k_{i}$ refers to the *i*-th assigned code for each topics
2. Based on the topics listed in first two columns of the Table A1, we then create a third column as shown in Appendix A, where the elements constitute the set $Y=\{z_{ij}:z_{ij}\in X,1<i<N,1<j<M,M\leq N-1\}$: That is, topic $k_{i}$ may contain up to *M* direct connections outlined in the corresponding text being mapped, M∈N. The elements of set *Y* therefore represent distinct topics in *X* which are related to $k_{i}$.

**Figure 2:**
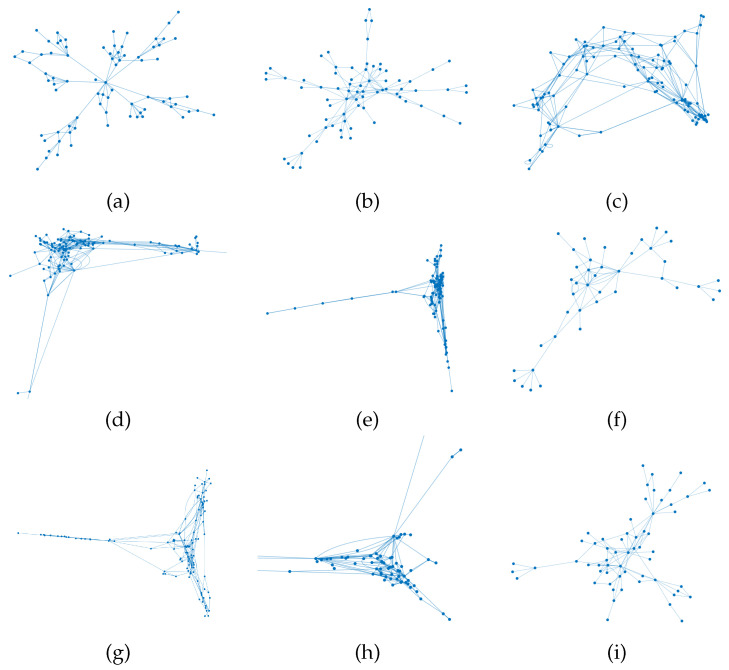
Network structure of the Pathways Combined curriculum. The various panels represent different books, namely: (**a**) Abramson (**b**) Blitzer, (**c**) CME, (**d**) COMAP, (**e**) Faires, (**f**) Larson, (**g**) Pathways (combined), (**h**) Rockswold and (**i**) Stewart. In cases (**d**,**h**), we provide a zoomed image to showcase the details of the connectivity.

Once the entire table is created, it can be represented as a network of nodes (topics) and edges (connections). Figure 2 gives examples of such a network, which is based on one of the various texts examined.

The model used here is built on a primarily backward referencing paradigm which suggests that topics in advanced chapters are built upon the foundations laid in earlier chapters. There could be exceptional cases where directionality is reversed but in a subject like mathematics, where courses (and in fact, the entire program) are built on prerequisite knowledge, one expects the arrow of knowledge acquisition to have a fixed orientation with respect to directionality. As a result of this implication, we do not see the need to use directed graphs for our analysis. The current model also uses unweighted graphs; weights would be akin to assessing the frequency of a particular connection. Our approach simply keeps track of the connections made, not whether they are repeated. While repetition is an essential aspect of learning, we feel that is a task best left to the instructor; repeating connections come with the side-effects of potentially increasing the size of the book and thereby the cost and also possibly detracting from other new connections that could be made. It could be argued that the Occam’s Razor principle of simplicity is apt for the curricular system.

### 3.3. Metrics Computed

Once represented as a graph, we can estimate several properties of such a graph which give us a glimpse of the underlying structure of the course and its potential to allow free and easy flow of ideas and foster new emergent understanding and creativity. By examining the network structure of various textbooks and pedagogical practices, we can help identify the kind of curricular plan that is likely to be most effective and creative. Specifically, we examine the following metrics [37,38]:

1. The **Degree Distribution** (DD) helps us ascertain that the ‘textbook network’ does indeed display a power law profile and hence the metrics typically associated with the analyses of such networks are meaningful in this context. The power law nature of such a network reveals that there is a specific structure to curriculum which is not random. The degree distribution of the network is given by the probability function
  (1) $$P\left(x\right)=c\;x^{-\alpha }$$
  where *c* is a constant, *x* denotes the degree of the node and α is the exponent which is determined through our computations.
2. **Clustering Coefficient** (CC) tells us about the average number of connections for each node, giving us a glimpse into the variety of ways a particular topic in precalculus can be understood. A fundamental assumption of the constructivist model of mathematics is the potential to make meaning. Therefore the greater the clustering coefficient, the more diverse the ways in which a concept can be comprehended depending on the particular background and proclivity of the student. The local clustering coefficient, denoted $C_{i}$, is commonly given by the expression:
  (2) $$C_{i}=\frac{3(\mathrm{number}\;\mathrm{of}\;\mathrm{triangles})}{\mathrm{number}\mathrm{of}\mathrm{connected}\mathrm{triples}}$$
  resulting in the average clustering coefficient for the network, $CC=\frac{1}{N}\sum_{1}^{N}C_{i}.$
3. **Average Path Length** (APL) tells us the average number of steps that must be taken to traverse between any two nodes. In the context of this study, the APL tells us about how efficiently one can move from one idea to another. It is particularly useful to strategize about how to resolve mathematical problems. A network possessing a low APL is preferable, since it makes explicit the links between concepts and provides a road-map to travel efficiently from one point to another. This, coupled with a high CC, makes for easy navigation between ideas and also increases the likelihood of exploring many possible ways to navigate between these ideas. The APL is given by the equation
  (3) $$APL=\frac{1}{N(N-1)}\sum_{i\neq j}d\left(V_{i},V_{j}\right)$$
  where $d(V_{i},V_{j})$ represents the path length from node *i* to node *j*.
4. **Hubs** (H) are nodes which have a large number of edges. The threshold number of edges to qualify to be a hub, in general, is determined by the nature of the problem itself. We use the minimum number of chapters from all the texts examined to decide a threshold to qualify for a hub. This number turns out to be 6 based on the book by Faires [30].

In addition, other general characteristics of the books are also examined such as the ratio of nodes to edges, and the specific topics that qualify as hubs across the different books.

## 4. Results

### 4.1. Power Law Distribution

Throughout the effort to meaningfully analyze quantitative patterns exhibited through these network representations of course-oriented textbooks, it initially became visually evident via generation of node-oriented degree distributions relative to frequency that there is a natural power law exhibited through these connections. Power laws occur in many natural and man-made systems, such as the frequency of particular words in a human language and population density ([39]). With these extensive applications to social structures, a natural hypothesis to generate is that the frequency of connections formed between topics in a given text will follow this relationship. While ’power law’ acts as a blanket term for many different types of data distributions, we in particular seek a pure power law following the aforementioned probability distribution of the form $P\left(x\right)=c\;x^{-\alpha }$, where *c* is a constant and α is a shape parameter generally provided as a positive real number greater than one, with *x* = the number of directly connected edges to a provided node, and P(x) = frequency of nodes possessing this connectivity index. The implication of this distribution is that there tends to be a high frequency of low-level connectivity; that is, many nodes have a low number of directly connected edges, while few nodes have a very high connectivity relative to other nodes in the network. This pattern indicates that superior connectivity is not necessarily expected to be the standard, and only the most pertinent topics introduced in the text are intended to be assigned as a common theme by which other introduced subject matter acts as a vehicle for consistent scaffolding of the main ideas behind each chapter and/or section.

It is worth noting that the associated parameters output by MATLAB, the primary computer algebra system we employed to carry out many relevant computations, generates parameters labeled *k* and σ. However, we are able to obtain a simple transformation in order to rewrite our power law distribution in the aforementioned format, where $c=k/\sigma^{2}$ and α=(k+1)/k. This transformation simply corresponds to the assumed format that MATLB operates within when generating fitted distributions belonging to the family of power law probability functions.

In a pure sense, a power law only fits the definition of a probability distribution provided that $x>x_{min}$, where we can meaningfully interpret $x_{min}$ to be equal to one (if $x_{min}=0$, we have no connections). Initially, many modern computer algebra systems will generate a fitted power law by testing the tail behavior of the input histogram plot, and use this information as a metric to decide whether or not the relationship is that of a Generalized Pareto, or should be reasonably approximated using a polynomial function with a finite tail. This assumption is unreasonable in this application because the meaning derived from this assignment is that a negative α-value and thus a positive exponent can equivalently be interpreted to mean that the range of the fitted tail following a power law fit is infinite, which certainly is not expected to be the case when dealing with data based on the confines of a printed textbook: Certainly, there exists a reasonable upper bound on the number of connections that can be formed. Thus, due to this decision, in our efforts we strictly fixed on fitting every text’s distribution to a pure power law with positive α values, yielding negative exponents, that fits our outlined probability mass function through analysis of a discrete number of links between various introduced topics.

However, a reasonable question to consider in this work is: ’How can one be sure that these fitted models, under these outlined parameters, are truly representative of the empirical model?’ While the data seems to draw visual cues towards the likelihood of belonging to the family of the power law distribution, this is certainly not sufficient to verify the accuracy of this hypothesis. While there is no doubt that every provided degree distribution is heavily right-skewed, there certainly exist various other distributions with arbitrary parameters that retain the same quintessential tail behavior known to belong to the exponential family: For example, a gamma distribution with α=θ=2.

To combat this ambiguity, the standard default method commonly sought, involves plotting the log of the frequency against the log of the relative ranking of empirical data and attempting to establish a linear relationship. However, this among other commonly used methods for analyzing power-law data, such as least-squares fitting, can produce substantially inaccurate estimates of parameters for power-law distributions, and even in cases where such methods return accurate answers they are still unsatisfactory because they give no indication of whether the data obey a power law at all [40]. Furthermore, inconsistent bin width of the inputted data further accentuated by sampling error-induced data noise can cause outlier values to be heavily magnified and obscure the true underlying distribution. It is for this reason that alternate methods become prominent to verify the integrity of the true nature underlying the data observed. Similar to the method outlined in the previously cited publication, which makes use of the Kolmogorov-Smirnov statistic, plotting the quantiles of the empirical distribution against the quantiles of the theorized distribution proves an effective choice as the expected density of the underlying function is preserved by comparison of ordered statistics, very much in the spirit of the aforementioned statistic. Thus, to ensure that our data fits well within the context of that of a pure power law, we make use of the quantile method of grouping our empirical data relative to the assumed underlying distribution to ensure homogeneous trends between uniform groupings. We can also verify the accuracy of the fitted distribution by verifying the CDF of a given network degree distribution also follows a power law with a less steep gradient, which again verifies the efficacy of this assumption.

**Figure 3:**
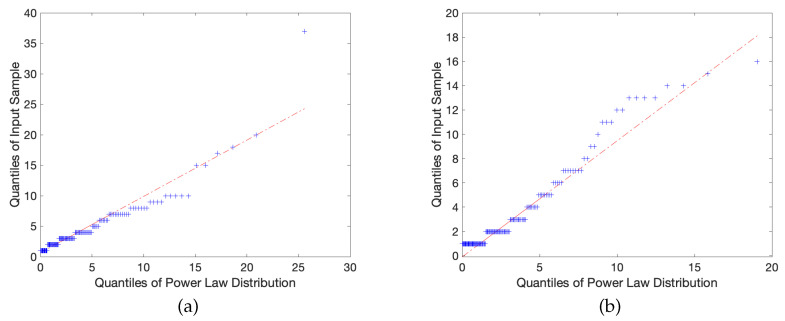
A Q-Q plot for the (**a**) CME and (**b**) Pathways books shows a linear profile indicating the strong possibility of a power law distribution.

**Figure 4:**
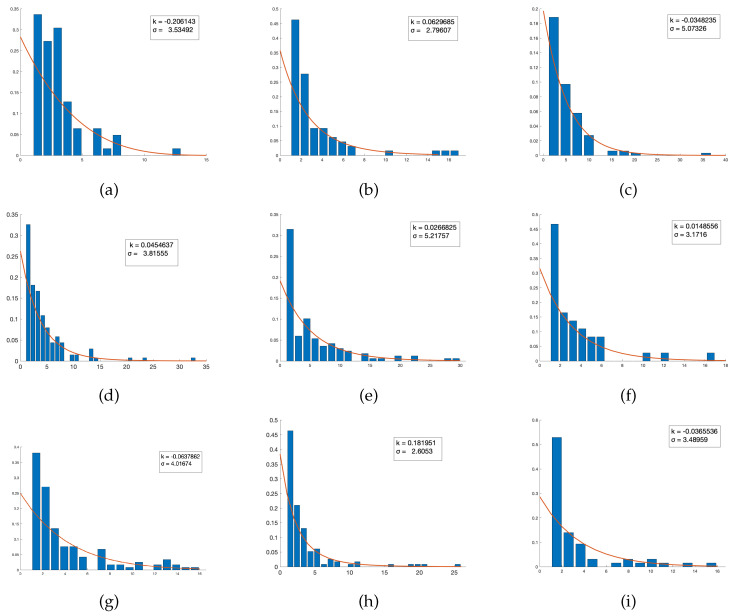
This graph shows the degree distribution for the various textbooks along with the power law fit superimposed. The various panels represent different books, namely: (**a**) Ambramson (**b**) Blitzer, (**c**) CME, (**d**) COMAP, (**e**) Faires, (**f**) Larson, (**g**) Pathways (combined), (**h**) Rockswold and (**i**) Stewart.

These statistical corroborations and power law distribution fits can be observed in Figure 3 and Figure 4 below:

In a mathematically rigorous sense, the construction of these quantile plots is generated by taking the cumulative distribution functions of our empirical data set *F* and our hypothesized power law distribution *G*, and obtaining their respective inverses $F^{-1},G^{-1}$, aka the quantile functions, and plotting them on the *x* and *y* axes. In this process, the *k*-th quantile of *F* is compared to the *k*-th quantile of *G*, where meaningful choices of *k* are usually specified by splitting into quartile iterations. While many quantile spacing options exist, a natural choice to employ in our study was, given *n* discrete samples, to space all *k* quantiles along the uniform distribution k/(n+1), k=1,2,…,n, in order to prevent distortion or uneven spacing of sample values. In a general sense, the corresponding measure of accuracy is determined by the size of the sample itself, allowing the model to self-adjust to prevent unearned weight from extreme outlier values.

If the two data sets are identical, we obtain overlapping plots along the line $F_{k}=G_{k}$. However, in the pursuit of collecting real-world data, we accept some reasonable tolerance of error; that is, we say that $|F^{-1}-G^{-1}|\;<\epsilon$ for some ϵ close to zero. In the examples provided, we see that the data sets follow each other closely and, despite local regions of fluctuation, the two appear highly correlated and follow a reasonably linear relationship. The theoretical desired result here is that the output produces a perfect linear relationship, in this case preserving mathematical integrity because outliers are no longer heavily skewing the data, of which is the primary concern for every other previously mentioned problematic schema. As observed in the above figures, a sample of quantile plots are provided in which the observed trend largely consists of a reasonably constant gradient, verifying that the underlying hypothesized power law is indeed the true distribution.

This discovery is particularly compelling because the vast majority of scaling parameters observed in power laws tend to lie in the interval (2,3). However, there exist observed parameters in our sample space that go as high as nearly 70, with a significant amount that are approximately 20. What does this tell us? Because highly negative exponents cause larger values of *x* to decay more rapidly, a distribution in which α is estimated to be extraneously positive has an uncharacteristically thinner tail relative to other observed samples. Thus, the data is comparatively more right-skewed, which tells us that the degrees in that network tend to be significantly lower and thus less highly connected than texts with shape parameters falling closer to the expected range. With respect to patterns observed in other metrics, it was observed that nearly all the measured texts held Average Path Lengths between three and four, meaning that these successful texts all share the feature that no more than four topics are expected to exist between any two chosen topics in order to present an effective pedagogical model by which an instructor may follow to form a strong basis upon which further, deeper connections may be introduced if deemed necessary for a particular person’s needs. Moreover, Average Local Clustering Coefficients for texts with particular low average path length tend to be in or near the interval (0.3,0.4), allowing one to draw the conclusion that any given topic’s close neighbors should remain fairly easily accessible in a sampling of curriculum about 40% of the time. However, does this imply by itself that a given text is objectively superior to another? Due to the complex nature of the connectivity of curriculum, a more accurate picture is generated by considering the ramifications of all observed metrics relative to one another, and identifying strong suits based on a particular instructor’s or student body’s particular needs.

While one might be inclined to raise suspicion with respect to the texts that retain extremely negative exponents when fitted to a power law, a reasonable question arises: Do there exist distributions that are better suited for these texts? Due to the nature of the plotted DD of each text, and the property that all are heavily right-skewed, another possible distribution that could capture this behavior is that of the exponential distribution (relative to the exponential distribution, we obtained a single scale parameter μ equal to the mean and the standard deviation of the fitted model). To test this hypothesis that a power law may not hold, an exponential fit was performed on the DD of the two texts with the lowest exponents (in this case, the Rockswold text and the Larson text), and quantile plots were generated to test the empirical distributions against the hypothetical fitted distributions.

The results reveal similar results for both tested distributions: There is a reasonably proper fit for approximately the first two quartiles, but beyond that exists significant outlier behavior in both the power law and exponential fits. This is indicative of two possible conclusions: The first is that extreme outliers are reasonably expected as they indicate an unexpectedly large number of connections being formed for few topics, thus potentially inducing saturation of content. The second possible conclusion is that these texts in particular are not reasonably fitted by either distribution. However, in either scenario, the conclusion appears valid as these tests were performed on texts that exhibited considerably lower metrics than other textbooks sampled. So, if the power law fit is a reasonable model, the fact that these course frameworks do not follow the proper trend indicates that, by inherent construction, perhaps there are elements of these texts that do not follow what might be considered more desirable pedagogical techniques, and thus are not well-captured by a distribution that properly represent texts with considerably improved metrics.

To test the fit of a model that is likely inappropriate relative to our understanding of pedagogical connectivity, we include a QQ-plot of a fitted normal distribution for both aforementioned texts. Our qualitative results indicate that this is, in fact, a poor choice of model; the relative quantiles are highly mismatched and deviate heavily from any sort of linear relationship, which provides reasonable insight into the notion that, should there exist more suitable models other than those we tested and hypothesized, we can expect that they are at least similar in quality to the power law and exponential, likely belonging to the family of right-skewed distributions.

While any given text might serve one educator or student’s goal more than another, and perhaps it is possible that a textbook might serve a certain environment well with the supplementation of content by a good teacher, the observation of these traits when compared across all texts permeates the suspicion that successful texts should exhibit similar connectivity trends, and should be actively considered when choosing an appropriate text to guide one’s lessons.

**Figure 5:**
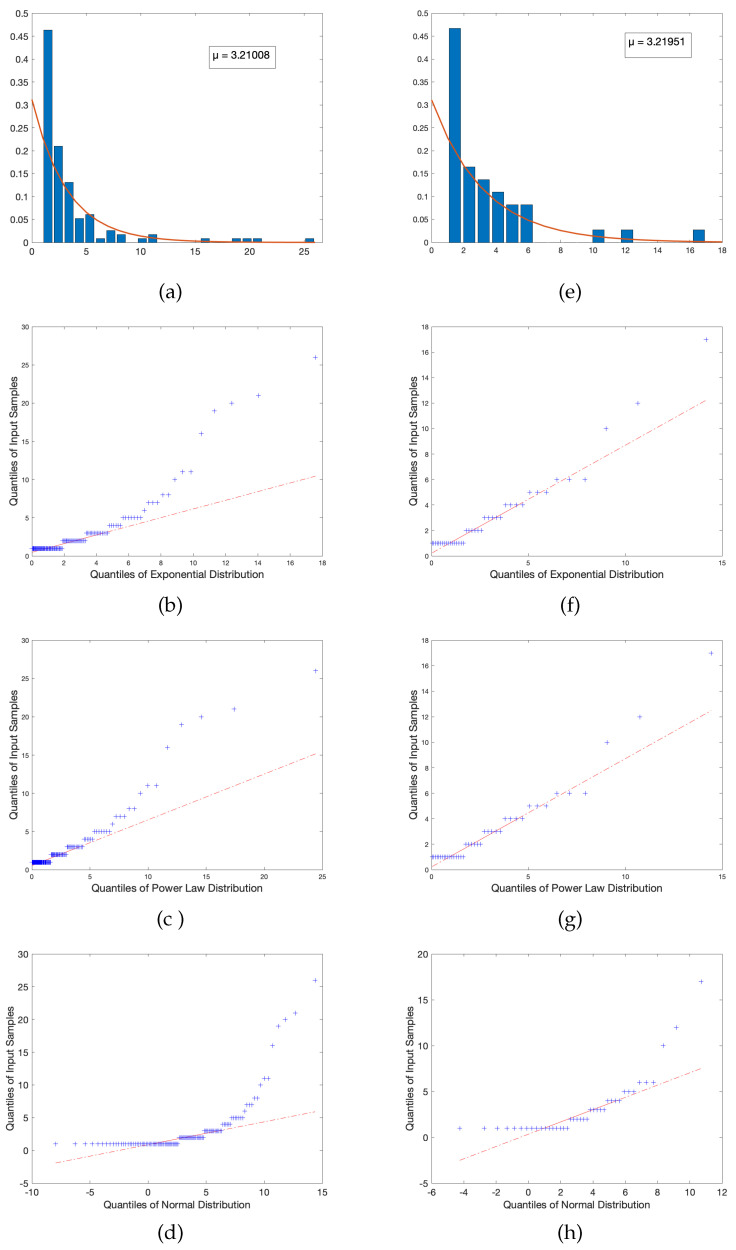
A visual display of the degree distribution behavior of two texts with outlier power law parameter values. (**a**–**d**) display the DD and all aforementioned Q-Q plots of Rockswold, while (**e**–**h**) display the same information for Larson.

A plot of the aforementioned selected texts’ DD fitted to an exponential distribution, as well as their respective quantile plots for every tested distribution, can be observed in the Figure 5 below.

### 4.2. Comparing the Network Profiles

While a large portion of our observations regarding fitting distributions to a given text’s degree distribution relied on qualitative analysis of QQ-plots, we further verified these assumptions by conducting a $\chi^{2}$ goodness-of-fit test in which we seek to reject or fail to reject our null hypothesis $H_{0}$: The underlying distribution follows a power law. Following conventions with respect to this method, if our computed *p*-value is less than a predetermined α chosen based on our desired level of confidence, we reject $H_{0}$, and otherwise we fail to reject $H_{0}$. The results were highly inspiring and promising alike: For every text included in the sample of this study, we found that for α=0.05 we fail to reject $H_{0}$ and thus uncover compelling evidence that a power law is a suitable model to describe the nature of the layout of topic relavance in a text of similar quality to those in our sample. For the two texts that appeared to have outlier parameter values in the context of a power law fit, we conducted two additional $\chi^{2}$ tests in which our respective null hypotheses were that the underlying distribution follow (i.) an exponential distribution and (ii.) a normal distribution. In these tests, again with α=0.05, we found *p* to be significantly less than 0.05, with a range of [0.025,0.035] for the exponential case and a range of [0,0.026] for the normal case. This allows us to conclude that, amongst these three distributions, the choice of a power law is an exceedingly ideal case and appears to accurately capture the behavior of the underlying characteristics of such models, allowing further research efforts to seek out desirable properties based on these observable patterns. These *p*-values pertaining to the power law case for each textbook can be observed in the Table 2 below.

The primary significance that emerges with assigning a fitted distribution is the comparison of meaningful metrics relative to other values extracted. A particularly clear pattern we have observed is that the exponent of the fitted distribution and the average path length generally exhibit a positively correlated relationship. One can interpret this to mean that texts with more rapidly-decaying tails, and thus more novel connections, tend to be more locally clustered by means of edge connectivity. While this might seem immediately intuitive, this certainly should not be expected to be the standard case. For example, it is plausible that a given network have low individual connectivity across nodes, yet the global trend is very spread out and disconnected, visually appearing as a sort of extended ‘branched-out’ graph. To couple rapidly-decaying fitted models with efficient traversing between topics implies that while individual ideas present in the text are not applied heavily to each other in a global sense, there is highly efficient ‘meaning-making’ in these course templates permeated by the fact that presented topics are introduced in a highly intentional manner. Low connectivity tells us that a given portion of the intended syllabus is isolated in terms of extension of application, yet easily accessible through other units indicated by minimal average path length.

Another non-trivial observation is that networks with a greater number of nodes tend to produce higher average local clustering coefficients. While this can naively be thought of as a symptom of greater sampling frequency, it is also significant to consider that a significantly greater percentage of hubs arises in higher-density networks. Thus, not only are these texts accessing a greater breadth of information, but they seem to be doing so with greater depth and significance upon consideration of topics introduced as a result. While we continue to question what makes certain texts (and therefore courses) more effective than others, it seems that a meaningful effort to bridge the gap between instructor convenience and student accessibility both play a clear role in the success of retention and connectivity as one progresses through this course, or even perhaps future courses that call on this material. While it is unsurprising that networks displaying high average local clustering coefficients tend to yield greater concentration of hubs, to do so amidst a rich curriculum (both in density and meaning) speak to the ability to continue forging meaning in the classroom, and continually placing this effort at the forefront of future textbook and classroom design.

## 5. Network Resilience Analysis

While the metrics we have computed thus far seem to have some tendencies to cluster relative to one another, against what basis are we comparing these values? Intuitively, one valid choice for an attempt to standardize these metrics might be what one could consider an ‘optimal curriculum’. A possible definition for an optimal curriculum would be one in which every observed connection across all sampled texts is made, including all aspects of what independent authors and publishers consider to be paramount for the discussion and implementation of precalculus in a course. With this in mind, we constructed such a ‘union graph’ in which we take the union of all encoded textbooks, yielding a network consisting of 697 total unique nodes and 1614 unique edges. We then computed all relevant metrics that we wished to compare the sampled texts to establish a yardstick against which all included textbooks could be compared to measure relative effectiveness in the context of a maximally efficient and inclusive course design. Upon such computation, we found that the APL for this union graph was 5.1108, and the CC was 0.3166. While these both measure reasonably within the range of corresponding values for each individual text, one may draw the reasonable conclusion that any text that retains a lower APL than the union is able to traverse introduced topics more efficiently than a course in which all possible reasonable connections might be made; similarly, any text with a greater CC than the union retains higher relevance to its relative neighbors, indicating a strong proximity to closely clustered subject matter and thus a higher potential for students to develop significant meaning across the course by observing rich connections across a large proportion of material.

**Figure 6:**
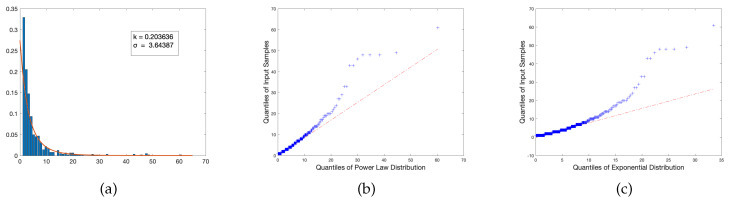
A visual demonstration of the trends observed in the union of all sampled texts, where (**a**) is the degree distribution fitted to a power law with relevant parameters, (**b**) is the Q-Q plot of the union with respect to a power law fit, and (**c**) with respect to an exponential distribution fit.

Additionally, we tested via quantile plots whether or not the union graph’s DD tended to follow a power law distribution or an exponential distribution, as heavily indicated by its right skew and tail behavior. The quantile plots revealed that while both retain significant outliers until about the sixtieth percentile, the power law has a slightly better relative regression to the power law fit versus the exponential; although the exponential has a greater density of sample values within the first quartile, this behavior becomes much more deviated heading towards the median than the former, having significantly more negative concavity than the power law’s quantile plot. With the reasonable assumption that, in practice, information provided and discussed early on in the trajectory of a course is typically better absorbed by students in the classroom, this potentially indicates that a significant presence of topics introduced that fall below the standard for a hub makes for an effective lesson template, preventing the potential saturation of information as it is further developed. The quantile plots of both fitted distributions, as well as the union graph’s degree distribution fitted to a power law, are provided below in Figure 6.

### 5.1. Stochastic Network Simulations

While the intended process of encoding textbooks in a network format is designed to be as purely objective as possible, the very presence of human interaction lends itself to degrees of subjectivity. For example, given the sentence, “This function has a constant rate of change, which we had identified previously”, most would likely agree that there is an inherent connection displayed here between ‘function’ and ‘constant rate of change’. However, would this warrant a further connection to ‘linear functions’? How do we resolve ‘we had identified previously’: Perhaps by increasing the edge weight? All these considerations are relatively proper and valid, but reveals the insight that we as independent human beings are truly incapable of a unified, objective motive by which we can perform the same automated process and consistently yield the same results. Even if we were to encode texts using a word recognition algorithm, the fact that a human had to write the program could itself lead to implicit bias that would cause differing results if written by two different people. So, how does one overcome this obstacle?

Our solution to this problem involves a series of stochastic simulations to test the sensitivity of dispersion across relevant metrics to this work. In the anticipated outcome that any given person could possibly miss any given number of connections, a MATLAB script was written that randomly selects a predetermined number of edges (where n= # of randomly selected edges removed) from the union graph and removes them from the network. After each iteration, the average path length and clustering coefficient are computed and printed to be stored for reference and comparison. Because the union graph contains 1614 edges, we started with a test case of removing five edges, and from there moved up as percentages of the total amount: n=80 edges as approximately five percent of the total network, n=160 as approximately ten percent, n=320 as approximately twenty percent, and n=800 as approximately fifty percent. If this process yields an isolated node, we simply remove it from the graph, as this would yield an infinite average path length and a similarly unreasonable clustering coefficient. After running each case for fifty iterations, we computed the mean APL and CC, as well as their respective standard deviations, in order to see how percentage of removed connections impacts the efficiency with which one may navigate a course based on attempting to maximize all conceived links.

### 5.2. Simulation Results

Upon analysis of these metrics, we find that there is reasonably no significant additional error induced when five percent (n=80) of nodes are randomly removed; with a mean average path length of 5.7123, there is less than one additional topic one must introduce on average per selected topic (as will be noted in the explanation of the union graph metrics below) that is required to introduce in order to meaningfully move throughout an extensive union course in precalculus, which indicates that relatively little information is lost through this level of error. While the CC has lowered to 0.2871, this is simply an indication that isolated nodes become less connected to their neighbors, and is reasonably farther away from being a complete graph; however, as we will discuss below, approaching a complete graph is not necessarily ideal in all desired scenarios.

**Figure 7:**
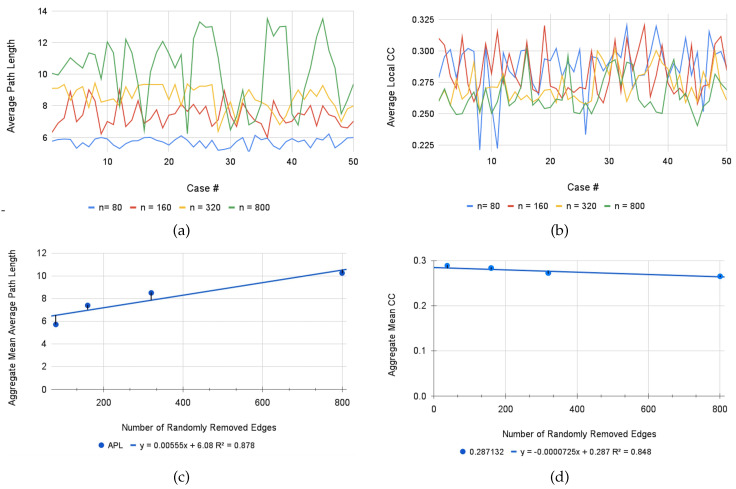
A visual display of both the variability of all fifty iterations for each value of n and the APL and CC metrics in (**a**,**b**), as well as a regression for the mean APL and CC across all iterations in (**c**,**d**).

There exists a significant separation from our initial case upon the removal of nearly fifty percent of all nodes (n=800), however even at this point, although there exists significant inconsistency of APL as indicated by a particularly high standard deviation of 2.1169, the mean APL resolves at 10.2547, which indicates a doubling of the standard required distance one needs to move through the course content. While increasing a given metric by one hundred percent is absolutely non-ideal, this communicates that even in the worst of circumstances in which half of a given curriculum’s connection are dissolved, one can still move through the subject matter with some degree of reasonability. Additionally observed in the case of n=800 is a mean CC of 0.2651, which further indicates that greater-degree iterations cause any given network to be further removed from the status of a complete graph; yet, compared to our basis as discussed below, we see that there is no significant loss of clustering, which yields the insight that overall traversing of course content is made reasonably more inefficient on a global scale, but there is still sufficient opportunity to easily travel across closely connected topics. Furthermore, it is absolutely reasonable to assume that a seasoned educator would scarcely commit an error past twenty percent removal, which is a strong reassurance that these metrics, and thus facilitation of precalculus subject content, retain significant resiliency in the face of pedagogical adversity. The metrics for each of these results can be observed in the Table 3 below. Additionally, displayed below in Figure 7 are plots of the aggregate APL and CC metrics for all iterated values of n.

## 6. Discussion

There are other visual assessment tools popularly used in education, including ‘Concept Maps’ [41] and ‘Y Charts’ [42,43]. Of these, concept maps come closest to the network idea that is discussed in this paper. Concept mapping has been popular in education for some time and were introduced by Joseph Novak in 1964 [44] to improve “students’ understanding of science concepts over a 12 year span of schooling” [41]. Since then, they have been popularly used in various educational settings and disciplines to enhance, synthesize information and as a reflective exercise. However, they are primarily used as a learning tool to gauge how students make meaning, i.e., in the context of learning, rather than how a curriculum is framed. Moreover, concept maps do not lend themselves to the kinds of computations presented in this paper since they are less focused on the kinds of details being addressed here. Trochim [45] rightly describes concept maps as lying at the cusp of ‘soft science’ and ‘hard art’; the current work can therefore be viewed as an exploration of the former, scientific aspect of this tool in the context of curricular design.

We recognize that books are written with different objectives in mind and can serve different audiences. We therefore make our interpretations with the understanding that the network metrics should be used to evaluate the affordances of a book. The teacher and the student can then realize, based on their respective goals, the appropriate book to use. However, we are also of the opinion that there are some objective traits captured by this analysis which allows for good pedagogy and enhanced opportunities for students to learn and make meaning. For a teacher or future author, this analysis can highlight, via the comparisons between different books, the kinds of interventions that need to be made to prepare students to better understand the material. If we were to take the union graph as an ideal network in terms of topics covered and connections made, then it is worthwhile examining how each of the books discussed here deviate on average from this ideal, null graph. Table 4 shows the networks ranked in terms of their closeness in traits to the union graph, as measured in terms of its room mean square deviation (RMSD), given by

(4) $$RMSD_{i}=\sqrt{\frac{{\left(CC_{0}-CC_{i}\right)}^{2}+{\left(APL_{0}-APL_{i}\right)}^{2}+{\left(PH_{0}-PH_{i}\right)}^{2}}{3}}$$

where $CC_{0}$, $APL_{0}$ and $PH_{0}$ represent the clustering coefficient, average path length and percentage of nodes with are hubs for the union network. The index i=1−9 corresponds to each book network. Of course, the rankings can change with depending on what metrics are valued and in this way, the analysis allows the instructors to bring their own standards to bear upon the decision of what network is closest to ideal.

What is the significance of providing rigorous mathematical models to present a standard model of educational practice? Through highly focused exploration of examining intentional connection of meaning between various topics explored across all sampled texts, the emergence of fairly predictable models indicates that neither random distributions of navigating the depths of curricula, nor a uniform presentation in which all topics maintain the same relative connectivity indices, are proper formats of effective and successful learning platforms. Instead, the data generated thus far is highly indicative of finding a meaningful balance between high frequency of low connectivity, providing temporary satellites which yield fruitful conclusions on a particular pedagogical path with respect to the curriculum as a whole, as well as low frequency of high connectivity, by which the instructor and student alike are able to extend meaning-making into many novel branches of thought that yield a unique individual interpretation within the context of the global pursuit of holistic and cohesive course understanding. It is therefore highly relevant that these well-regarded outlines of classroom instruction follow similar mathematical models, as this can allow future educators and textbook writers to establish a deeper analysis of the efficacy of intelligent design in conjunction with the student experience at the forefront of pedagogical innovation.

While each text involved in this study holds up reasonably well in the quantile plot analysis of the accuracy of fitting a power law distribution, there are a couple of texts that begin to lose their linear trend when approaching outlier nodes. Upon deeper examination, a critical question to consider is: What factors seem to contribute to the encoding of particular texts who, when compared to the quantiles of their corresponding theoretical distribution, do not seem to fully exhibit a proper power law?

Texts whose empirical data plots appear slightly nonlinear when placed in a Q-Q plot (relative to other well-regressed texts, as provided previously) tend to have highly negative exponents, with low hub percentage despite reasonably effective path lengths and clustering coefficients. Because our data seems to suggest that highly rated effective texts, upon aggregation of relevant metrics, tend to exhibit strong power law behavior, and a highly negative exponent itself indicates a rapidly decaying tail and thus less extremely well-connected nodes, a lack of highly connective topics appears to be a key component of the observation of patterns within effective pedagogical models of curriculum. That is, without a high presence of hubs, deeper levels of meaning are no longer as effectively extracted from the content, yielding much more novel connectivity and thus preventing the paramount experience of meaningful student connection with the mathematics.

When students are able to observe vast connections across many topics and even areas of their own lives, we believe that the classroom experience becomes a much more conducive environment to retaining information, especially as a given student moves through future mathematics courses that will assume a proper and well-tethered understanding of the material. Therefore, if a power law seems to not hold, one might hypothesize that this is likely a symptom of less effective curriculum design (this is a point that deserves further attention through a thorough analysis and comparison of various distribution functions, the task being to find the optimal one that fits the entire DD profile). However, it is relevant to note that every plot in this sample is highly linear through the seventy fifth percentile, providing the notion that outliers in the data (highly connected nodes) are a critical element of composite distribution trending. While their influence is undermined in other novel tests (such as log plots, as previously discussed), they are appropriately stated and stressed in their ability to display the deviations in correlation between the empirical and theoretical relationship across the aggregate collection of sampled coursework models.

Aside from the analysis of the generated degree distributions, the networks themselves yield incredibly valuable insights into not only the extent to which global connectivity is achieved, but also how local behavior provides a direct lens into higher-order navigation of scaffolding ideas and collective classroom procedure. Two of the most valuable metrics in this discussion that emerged, despite the consideration of many others, are average path length (APL) and average local clustering coefficient (CC). Nearly every text sampled retained an APL between 3.0 and 4.0, indicating that efficient navigation of precalculus discourse should keep local topics well-connected, requiring no more than a few select topics to serve as the roadmap between any given ideas. Surely, with our minds focused on the educational ramifications, this feels to be an appropriate conclusion, as the goal in such a foundational mathematics course is to consistently relay the notion that the depth of the curriculum is circumvented by the relevance of all chosen topics with respect to one another. A successful classroom experience is not one of isolation; it is one of intention, retention, and ever furthering the endeavor to highlight the reasons behind studying and analyzing functions, relations, and all that which makes up a proper course. Similarly, most CC values tend to lie between 0.3 and 0.4, indicating that any given topic should be connected to between 30% and 40% of its direct neighbors, again easing the path each student takes in their endurance to tether their interpretation with all previously learned and future material.

The minimum requirement of six directly connected nodes to qualify as a hub was chosen through the observation that the minimum number of chapters provided across all texts examined was itself six, providing the initial conception that we are indicating a topic that has the potential to reach across the minimum span of any given network. After implementing this criterion and generating each respective network, it was discovered that there are only two common hubs across every single textbook sampled: ‘Functions and Relations’ (F/R) and ‘Polynomial Functions’ (PF). (see Appendix B and Appendix C for a comprehensive list of all hubs across all the texts analyzed and a book by book breakdown) This deepens our pursuit of answering a fundamental question that permeates the mathematical community as a whole: What is the purpose of precalculus as its own course? Does it simply serve as a precursor to calculus, or is there deeper intention behind the syllabus? This pattern properly provides a reasonable response in that precalculus is the study of functions and their individual and collective properties. In everyday life, relations bring about our human desire to establish meaning behind call-and-response, input and output relationships. We study mathematics because we desire the understanding of the world around us: Similarly, students and instructors study precalculus because it refines our rigor and analytical ability to effectively communicate the similarities and differences in classes of relationships and experiences through the natural world, and perhaps the unnatural that we simply have yet to understand. There serves no better bridge between the world of entry-level and high-level mathematics than elucidating the meaning of functions and polynomials, as these serve as the backbone of analysis, the construction of meaning-making, and the ever-driving wandering through the question, ‘How does this connect to our understanding, and therefore connect understanding itself’?

The second law of thermodynamics, which dictates that the very act of using available energy for work produces non decreasing amounts of entropy, is as applicable to this setting as in natural phenomena [23]. We recognize that the very act of mapping a curriculum, produces losses; our network metrics cannot possibly capture what transpires within a classroom. The role of the teacher, as a synthesizer, story teller, bridge -builder and inspirer goes unaddressed. There are always qualitative aspects of a system which evade the grasp of a purely quantitative analysis. A novice teacher, who uses a book literally, will benefit directly from the analysis presented here. A seasoned teacher who uses the textbook only as a guide and adds their own unique perspective is already doing what we hope teachers would, i.e., build bridges between topics and allow students to journey through the subject in a variety of ways. In the case of such teachers as well, such a mapping can give a glimpse of the strengths and connections that already exist and the lost opportunities that can be built upon.

## 7. Conclusions

In summary, this paper uses tools from network theory to discuss the nature of connectivity in education, presented through the example of precalculus textbooks. While the primary focus surrounding much of this paper has been devoted to the complex nature of a mathematics curriculum in general, there are specific takeaways for the power of network theory to deal with issues pertaining to education and specifically, curriculum design. The analysis would also be helpful to educators interested in the instruction of precalculus.

The Table 4 suggests networks that perform well in accordance with Equation (4), which is subject to the preferences of the authors. In making this choice we are implicitly suggesting that: (a) An optimal APL allows the student to arrive at complex ideas along a reasonably short path. While a very large APL is reflective of long-windedness, a very short one can be dismissive of the complexity of the topic and skim over ideas that need elaboration, thus there exists a reasonable interval over which this metric must lie. (b) The CC needs to be sufficiently high, allowing students to find different ways to recall the course content. (c) The number of hubs must be reasonable, i.e., the text does not take the extreme attitudes of triviality or weight. This perspective is reflected in the power law exponent, where a highly positive α would contain a predominantly large number of topics with few connections and an α close to 0 would be reflective of an attitude of uniformity, which neglects complexity and students’ struggle with advanced topics.

While all books cover a diverse set of topics, they are structured differently and with different intentions. If we were forced to make a recommendation for an optimal book, we suggest one where the CC, APL and PH are closest to an ideal (or null) network, in our case exemplified by the union graph and shown in Table 4. We are hopeful that the development of this thinking will continue to shine light on the plight of the mathematical explorer, and how intentional design can improve the aggregation of successful curriculum across the subject, the discipline, and the world. We are well aware of the various shortcomings of the study, including a more in-depth study of the distribution profile and other pertinent network characteristics which might be useful. We hope to address them in future papers.

## Figures and Tables

**Table 1:** Detailed description of Precalculus textbooks analyzed in this paper.

Text (Reference)	First Author	Edition	Year	Publisher
[25]	Abramson	1st	2017	OpenStax
[26]	Blitzer	5th	2013	Pearson
[27]	Carlson	8th	2020	MacMillan
[28]	CME	-	2013	-
[29]	COMAP	-	2002	W. H. Freeman
[30]	Faires	5th	2011	Cengage
[31]	Larson	3rd	2000	Houghton Mifflin
[32]	Rockwold	4th	2010	Pearson
[33]	Stewart	6th	2012	Cengage
[34]	Sullivan	11th	2020	Pearson

**Table 2:** The results of the network analysis for all texts are summarized in this table. Quantities computed include the average path length, clustering coefficient, number of hubs, percentage of nodes that are hubs, number of edges and nodes for each text.

Book	APL	CC	% H	# H	Edges/Nodes	Mean Edges per Node	Power-Law Exponent	*p*-Value
Abramson	19.0047	0.2662	11.54	9	116/78	1.487	−5.851	0.14
Blitzer	3.7652	0.0481	12.33	9	109/73	1.493	−16.881	0.16
Carlson	3.9001	0.2725	21.83	31	268/142	1.887	−16.6774	0.07
Stewart	3.361	0.0417	15.00	9	101/60	1.683	−28.357	0.06
Faires	3.2133	0.3414	35.25	43	327/122	2.680	−38.478	0.84
CME	3.4179	0.4152	32.03	41	314/128	2.453	−29.716	0.06
Larson	3.6915	0.156	14.63	6	66/41	1.610	−68.315	0.051
COMAP	3.6908	0.3397	21.19	32	302/151	2	−22.996	0.22
Rockswold	3.4128	0.2815	11.76	14	192/119	1.613	−55.960	0.07

**Table 3:** The results of the stochastic network simulation analysis for the union of all texts are summarized in this table. Quantities computed include the average path length, clustering coefficient, and error metrics relative to the original union graph.

No. of Removed Nodes, n	n = 5	n = 80	n = 160	n = 320	n = 800
Percent Equivalent of Total Edges	0.3097	4.9566	9.9132	19.8265	49.5662
Absolute Percent Error in APL	0.0666	11.7704	44.7580	66.4542	100.6488
Absolute Percent Error in CC	0.8559	9.3076	10.4080	13.9279	16.2400
Mean APL	5.1142	5.7123	7.3982	9.9291	12.9921
Standard Deviation of APL	0.0029	0.2820	0.7283	0.7835	2.1169
Mean CC	0.3138	0.2871	0.2836	0.2725	0.2651
Standard Deviation of CC	0.0028	0.0210	0.0190	0.0132	0.0146

**Table 4:** A ranking of texts on the basis of their root mean square deviation from the union graph.

Rank	Network	Composite RMSD	Rank of Graph Size Based on # of Nodes
1	Pathways	1.2989	2
2	COMAP	1.7228	1
3	CME	4.8429	3
4	Stewart	5.1918	8
5	Larson	5.3651	9
6	Blitzer	6.6795	7
7	Faires	6.6892	4
8	Rockswold	7.0268	5
9	Abramson	10.7050	6

## Data Availability

All data generated through this work has been reported in this paper. Those interested in discussing potential for additional data generation through this work can contact us via email.

## Appendix A. Network Construction

The table below shows the coding for the topics identified in each text and also the connections between topics (column 3). We demonstrate the text to network mapping for the Stewart text as an example. A similar procedure is used to encode and map the other texts.

**Table A1:** This table provides the codes and connected topics in the text by Stewart [33].

Topic Name	Code	Directly Connected Topics
Polynomial Functions	PF	RATZEROTHM, RF, REMAINALG, DIVALG, FACTTHM, GRAPHTECH, UPLOWBOUND, QUAD, LINEARFUNC, NONLIN, INEQ, F/R, DESCARTES,
Rational Zero Theorem	RATZEROTHM	PF
Rational Functions	RF	PF, GRAPH, MOD, PARTFRAC, LINEARFUNC, NONLIN, INEQ, F/R
Transformations	TRANS	GRAPH
Graphs	GRAPH	RF, TRANS, ASYM, MOD, EXP, LOG, EQN, PERIODICFUNC, LINEARFUNC, SYSLIN, LINPROG
Asymptotes	ASYM	GRAPH
Modeling	MOD	RF, GRAPH, EXP, LOG, EQN, PERIODICFUNC, LAWS, TRIGRAT, MATRIX, SYSLIN, HYPERBOLA, ELLIPSE, PARABOLA, SEQUENCES, BIN, RECURS
Partial Fractions	PARTFRAC	RF
Remainder Algorithm	REMAINALG	PF, SYNTH
Synthetic Division	SYNTH	REMAINALG, DIVALG
Division Algorithm	DIVALG	PF, SYNTH, LONGDIV
Long Division	LONGDIV	DIVALG
Factor Theorem	FACTTHM	PF
Intermediate Value Theorem	IVT	GRAPHTECH
Graphing with Technology	GRAPHTECH	PF, IVT
Descartes’ Rule	DESCARTES	PF
Upper and Lower Bounds	UPLOWBOUND	PF
Quadratic Functions	QUAD	PF, QUADGRAPHING, OPTIM
Graphing Quadratics	QUADGRAPHING	QUAD
Optimization	OPTIM	QUAD
Exponential Functions	EXP	GRAPH, MOD, LOG, EQN, NAT, F/R
Logarithms	LOG	GRAPH, MOD, EXP, EQN, NAT, COM, LIQU, F/R
Equation Representations	EQN	GRAPH, MOD, EXP, LOG, TRIG, IDENT, HYPERBOLA, ELLIPSE, PARABOLA, POLAR
Natural Log	NAT	LOG, EXP
Common Log	COM	LOG
Application: Liquids	LIQU	LOG
Trigonometry	TRIG	EXP, LOG
Right Triangle Trig	RTTRI	LOG
Unit Circle	UNITCIRCLE	LOG
Trig Identities	IDENT	EQN, UNITCIRCLE
Periodic Functions	PERIODICFUNC	MOD, RTTRI
Inverses	INVERSE	RTTRI
Trig Laws	LAWS	MOD, RTTRI
Trig Ratios	TRIGRAT	MOD, RTTRI
Matrices	MATRIX	MOD, METHODS, SYSOFEQNS
Methods of Evaluating Systems	METHODS	MATRICES, DET, CRAMER, GAUSSJORD
Systems of Equations	SYSOFEQNS	MATRIX, LINEARFUNC
Determinants	DET	METHODS
Cramer’s Rule	CRAMER	METHODS
Gauss-Jordan	GAUSSJORD	METHODS
Linear Functions	LINEARFUNC	PF, RF, GRAPH, SYSOFEQNS, F/R
Non-Linear Functions	NONLIN	PF, RF, SYSLIN, F/R
Systems of Non-Linear Eqns	SYSNLIN	GRAPH, MOD, NONLIN, INEQ
Inequalities	INEQ	PF, RF, SYSLIN, F/R
Linear Programming	LINPROG	GRAPH
Conic	CONIC	HYPERBOLA, ELLIPSE, PARABOLA, POLAR, F/R
Hyperbolas	HYPERBOLA	MOD, EQN, CONIC
Ellipses	ELLIPSE	MOD, EQN, CONIC
Parabolas	PARABOLA	MOD, EQN, CONIC
Polar	POLAR	EQN, CONIC
Sequences	SEQUENCES	MOD, INDUCTION, BIN, RECURS, GEO, ARITH, SERIES, F/R
Proof by Induction	INDUCTION	PROOF, SEQUENCES
Binomial Theorem	BIN	MOD, SEQUENCES
Recursion	RECURS	MOD, SEQUENCES
Geometric Sequences	GEO	SEQUENCES, PARTIALSUMS
Arithmetic Sequences	ARITH	SEQUENCES, PARTIALSUMS
Series	SERIES	SEQUENCES, PARTIALSUMS
Partial Sums	PARTIALSUMS	GEO, ARITH, SERIES
Proofs	PROOF	INDUCTION
Functions and Relations	F/R	PF, RF, EXP, LOG, TRIG, LINEARFUNC, NONLIN, INEQ, CONIC, SEQUENCES

## Appendix B. Codes for Hubs in All Books

**Table A2:** This table provides the codes used for the hubs identified in all the books.

Hub Name	Code
Functions and Relations	F/R
Linear Functions	LF
Polynomial Functions	PF
Rational Functions	RF
Trig	TRIG
Conics	CONICS
Sequences	SEQUENCES
Limits	LIMITS
Series	SERIES
Graphing	GRAPH
Modeling	MOD
Inequalities	INEQ
Equations	EQN
Rate of Change	ROC
Average Speed	AS
Constant Rate of Change	CROC
Quadratic	QUAD
Composition	COMPOSITION
Function Notation	FUNCTNOT
Inverses	INV
Domain and Range	D/R
Exponential Function	EF
Growth and Decay	GROW/DEC
Transformations	TRANSF
Roots and End Behavior	ROOTS/EB
Circular Motion	CIRCMOT
Angle Measure	ANGMES
Cosine	COS
Sine	SIN
Right Triangle	RTTRI
Non-Right Triangles	NRTTRI
Average Rate of Change	AROC
Tangent	TAN
Change in Quantity	DELQ
Covariation	COV
Proportions	PROP
Box Activity	BOX
Percent Change	%DEL
Logarithm	LOG
Technology	TECH
Real Number Line	REALLN
X-Y Plane	XYPLANE
Applications	APPS
Distance Formula	DISTF
Pythagorean Theorem	PYTHTHM
Parabola	PARABOLA
Symmetry	SYMM
Calculus	CALC
Complex Numbers	COMPLEX
Periodic	PERIODIC
Cotangent	COT
Secant	SEC
Cosecant	COSEC
Trigonometric Identities	IDENTITIES
Sum and Difference Formulas	SUMDIFF
Hyperbola	HYPERBOLA
Ellipse	ELLIPSE
Circle	CIRC
Reflection	REFLECT
Quadratic Formula	QUADF
Polar Form	POLAR
Radians	RAD
Reciprocal	RECIP
Roots of Unity	ROOTUNITY
DeMoivre’s Theorem	DEMOIVRE
Secant Line	SECLINE
Slope	SLOPE
Euler’s Constant	E
Factorial	FACTORIAL
Tangent Line	TANLN
Combinatorics	COMBINATORICS
Mathematical Modeling	MATHMODEL
Dependent Variable	DV
Independent Variable	IV
Applications: Free Falling Objects	FALLINGOBJECTS
Oblique Triangles	OBLIQUE
Vectors	VECTORS
Differential and Difference Equations	DIFFEQ
Set Representations	SETS
Function Representations	REP
Role of Numbers and Quantity	NUMBERS
Permutations and Combinations	PERMUTCOMB
Counting Principles	COUNTPRINC
Probability	PROB
Binomial Expansion	BINEXP
Recursion	RECURS
Difference Tables	DIFFTAB
Tables	TAB
Proof	PROOF
Geometry	GEOM
Analytic Geometry	ANALGEOM
Coordinate Plane	COORDPL
Exponent Value	EXPVAL
Events	EVENTS
Area Under Curve	AREAUNDCURVE
Division Algorithm	DIVALG
Operations	OPER

## Appendix C. Hubs

The table below provides the hubs for each precalculus text studies in this paper.

**Table A3:** The hub topics for each textbook. Note that the threshold to qualify as a hub is based on the book by Faires which contains a minimum of six chapters.

Book	Hub Names
Abramson	RF, LF, PF, RF, TRIG, CONICS, SEQUENCES, LIMITS, SERIES
Blitzer	GRAPH, MOD, INEQ, RF, PF, EQN, CONICS, SERIES, F/R
Pathways	ROC, AS, CROC, QUAD, F/R, COMPOSITION, FUNCTNOT, INV, D/R, EF, GROW/DEC, PF, TRANSF, ROOTS/EB, RF, CIRCMOT, ANGMES, COS, SIN, RTTRI, NRTTRI
Stewart	PF, RF, GRAPH, MOD, LOG, EF, SEQUENCES, EQN R/F
Faires	R/F, LF, REALLN, XYPL, RF, APPLICATION, QUAD, D/R, GRAPH, TECH, PYTHTHM, TRANSF, PARABOLA, SYMM, CALC, INV, ROOTS, PF, COMPLEX, TRIG, SIN, COS, PERANG, ANG, TAN, COTAN, SEC, COSEC, IDENTITIES, SUMDIFF, RTTRI, EF, LOG, GROW/DEC, CONICS, ELLIPSE, HYPERBOLA, CIRC, REFLECT, QUADF, POLAR, DISTF, COMPOSITION
CME	TAN, SIN, COS, PYTHTHM, GRAPH, ANG, RADIANS, TRIG, CIRC, EQN, RECIPROCAL, COMPLEX, DEMOIVRE, PF, IDENTITIES, SUM, RF, SECLINE, SLOPE, EF, E, FACTORIAL, TANLN, COMBINATORICS, PERMUTCOMB, R/F, PROB, BINEXP, RECURS, DIFFTAB, TAB, PROOF, GEOM, ANALGEOM, COORDPL, EXPVAL, EVENTS, CALC, AREAUNDCURVE, COUNTPRINC, ROOTUNITY
Larson	DIVALG, PF, GRAPHS, MOD, EQNS, OPER
COMAP	TRANSF, LINEAR, GEOMETRY, PF, F/R, GRAPHS, MATHMODEL, TABLES, EQN, DV, IV, EXP, LOG, INVERSE, MODELING, FALLINGOBJECTS, TECHNOLOGY, COMPLEX, PERIODIC, COS, SIN, RADIAN, TAN, RTTRI, OBLIQUE, VECTORS, POLAR, MATRIX, ANALGEO, PARABOLA, COUNTINGPRINC, DIFFEQ
Rockswold	F/R, SETS, REP, GRAPHS, LINEAR, INEQ, NUMBERS, MODELS, ZERO, EQN, QUADRATIC, PF, DIVISION, RF
